# Sitagliptin prevents aggravation of endocrine and exocrine pancreatic damage in the Zucker Diabetic Fatty rat - focus on amelioration of metabolic profile and tissue cytoprotective properties

**DOI:** 10.1186/1758-5996-6-42

**Published:** 2014-03-20

**Authors:** Cristina Mega, Helena Vala, Paulo Rodrigues-Santos, Jorge Oliveira, Frederico Teixeira, Rosa Fernandes, Flávio Reis, Edite Teixeira de Lemos

**Affiliations:** 1 Laboratory of Pharmacology & Experimental Therapeutics, IBILI, Faculty of Medicine, Sub-Unit 1 (Polo III), University of Coimbra, 3000-548 Coimbra, Portugal; 2 ESAV, Polytechnic Institute of Viseu, Viseu, Portugal; 3 Center for Studies in Education, Technologies and Health (CI&DETS), Polytechnic Institute of Viseu, Viseu, Portugal; 4 Institute of Immunology, Faculty of Medicine, University of Coimbra, Coimbra, Portugal; 5 Immunology and Oncology Laboratory, Center for Neurosciences and Cell Biology, Coimbra, Portugal

**Keywords:** Type 2 diabetes, Endocrine and exocrine pancreas lesions, Sitagliptin, Cytoprotective properties, ZDF rat

## Abstract

**Background:**

The purpose of this study was to investigate some of the possible mechanisms underlying the protective effects of a dipeptidyl peptidase IV (DPP-IV) inhibitor, sitagliptin, on pancreatic tissue in an animal model of type 2 diabetes mellitus (T2DM), the Zucker Diabetic Fatty (ZDF) rat, focusing on glycaemic, insulinic and lipidic profiles, as well as, on apoptosis, inflammation, angiogenesis and proliferation mediators.

**Methods:**

Male obese diabetic ZDF (fa/fa) rats, aged 20 weeks, were treated with sitagliptin (10 mg/kg bw/day) during 6 weeks and compared to untreated diabetic and lean control littermates. Metabolic data was evaluated at the beginning and at the end of the treatment, including glycaemia, HbA1c, insulinaemia, HOMA-beta and TGs. Endocrine and exocrine pancreas lesions were assessed semiquantitatively by histopathological methods. Pancreas gene (mRNA) and protein expression of mediators of apoptotic machinery, inflammation and angiogenesis/proliferation (Bax, Bcl2, IL-1β, VEGF, PCNA and TRIB3) were analyzed by RT-qPCR and/or by immunohistochemistry.

**Results:**

Sitagliptin treatment for 6 weeks (between 20 and 26 week-old) was able to significantly (*p* < 0.001) ameliorate all the metabolic parameters, by preventing the increase in blood glucose and in serum TGs contents (16.54% and 37.63%, respectively, vs untreated), as well as, by preventing the decrease in serum insulin levels and in the functional beta cells capacity accessed via HOMA-beta index (156.28% and 191.74%, respectively, vs untreated). Sitagliptin-treated diabetic rats presented a reduced pancreas Bax/Bcl2 ratio, suggestive of an antiapoptotic effect; in addition, sitagliptin was able to completely reduce (*p* < 0.001) the pancreas overexpression of IL-1β and TRIB3 found in the untreated diabetic animals; and promoted a significant (*p* < 0.001) overexpression of VEGF and PCNA.

**Conclusion:**

In this animal model of obese T2DM (the ZDF rat), sitagliptin prevented β-cell dysfunction and evolution of pancreatic damage. The protective effects afforded by this DPP-IV inhibitor may derive from improvement of the metabolic profile (viewed by the amelioration of glucose and TGs levels and of insulin resistance) and from cytoprotective properties, such as antiapoptotic, anti-inflammatory, pro-angiogenic and pro-proliferative.

## Background

Type 2 diabetes mellitus (T2DM) prevalence and incidence is rapidly increasing worldwide; it is predicted, according to the latest estimates of the World Health Organization (WHO), that diabetes will be the 7th leading cause of death in 2030 [1]. T2DM is a chronic disease leading to macro- and microvascular complications, which results in severe illness and premature death, with elevated personal and economic costs [2,3].

The central features of T2DM are a defect in insulin resistance and/or insulin secretion, which lead to hyperglycaemia; disruption of the normal relationship between insulin sensitivity and pancreatic β-cell function is a hallmark of T2DM progression [4]. In fact, degeneration of Langerhans islets with β-cell loss is secondary to insulin resistance and is regarded as the most important lesion for progression of the disease [5,6]. As β-cell function declines, the impairment of insulin action becomes more important. Hyperglycaemia, *per se*, may have a detrimental effect on secretory function, − “glucotoxicity” –, which induces increased apoptosis in pancreatic islets; in addition, the abnormal lipid profile commonly observed in these subjects may be associated with functional impairment of the islet – “lipotoxicity” [5-8].

Current knowledge adds further complexity in the picture of T2DM pathogenesis by including the role of incretin hormones. Incretins [gastric inhibitory polypeptide (GIP) and glucagon-like peptide-1 (GLP-1)] are peptide hormones secreted in the gastrointestinal mucosa after meal ingestion. These are released in response to oral glucose intake and are able to attain physiological concentrations causing insulin release, which is called the “incretin effect” [9-12]. GLP-1 acts in a positive way on the β and δ cells, whereas GIP acts preferentially on α and β cells. These peptides are almost undetectable during fasting and exist only in high concentrations in the postprandial state, since they are rapidly metabolized [13] by the ubiquitous enzyme, dipeptidyl peptidase-IV (DPP-IV), to inactive metabolites, which are eliminated by urine [14]. The incretin effect is responsible for about 60% of the secretion of postprandial insulin, which is decreased in T2DM [15]. In these patients, the incretin effect is stifled, producing an “incretin defect”. This condition occurs as a result of reduced secretion of GLP-1, accelerated metabolism of GLP-1 and GIP, as well as defective response to both hormones, particularly to the insulinotropic effect of GIP [16,17]. The key mechanisms by which these factors exert their action on β-cells are not yet completely elucidated, but currently lie on metabolic processes such as apoptosis and inflammation, among others putatively involved.

Low-grade inflammation has been viewed as a major player in insulin resistance development and T2DM evolution; indeed, hyperglycaemia seems to induce the production of acute phase reactants from the adipose tissue, while obesity, present in many diabetic patients, is in itself, characterized as a state of low grade inflammation [18]. T2DM is found to display increased concentrations of C-reactive protein (CRP) and pro-inflammatory cytokines, such as tumor necrosis factor-α (TNF-α) and interleukins 1 and 6 (IL-1, IL-6), which are implicated in instigating metabolic insulin resistance [19]. However, it is not still clear which is the cause and/or the consequence. A recent study by Martin-Cordero et al. [20] using obese fa/fa obese Zucker rats confirmed the presence of augmented inflammatory markers (IL-6 and CRP) in metabolic syndrome (MS), together with increased noradrenaline contents; the authors postulate that those results may reflect a defective regulation of the negative inflammatory/stress feedback loop under those circumstances, suggesting that MS could be either the cause or the consequence of diabetes associated with obesity.

Additionally, although the loss of β -cell mass is not yet completely clarified, apoptosis seems to be involved, as previously observed in pancreas at autopsy and isolated islets from people with T2DM [21,22]. Based on these assumptions, it is becoming clear that T2DM management, namely by using pharmacological agents, must envision not only glycaemic control but also, and particularly, the mechanisms behind progression of pancreatic deterioration and underlying evolutional complications. In fact, T2DM therapeutics should be able to preserve β-cell mass as the mainstay of disease control, by addressing the mechanisms implicated in diabetic pathogenesis, including apoptosis, inflammation or even an added capacity for cell proliferation.

Enhancing the incretin effect is now a possible therapeutic target in T2DM, using GLP-1 analogues or DPP-IV inhibitors. Sitagliptin belongs to a class of oral antidiabetic drugs, the gliptins, which inhibit the enzyme DPP-IV that degrades incretins, prolonging the physiological actions of GLP-1 [23,24]. GLP-1, a prominent active compound of the incretin family, modulates many processes in pancreatic islet: it potentiates insulin synthesis and secretion [25], inhibits glucagon secretion [26], increases islet cell proliferation, and decreases cell apoptosis [27,28]. Our group has previously shown that sitagliptin is able to ameliorate dysmetabolism, insulin resistance, inflammation and oxidative stress in an animal model of T2DM, the Zucker Diabetic Fatty (ZDF) rat [29,30]. Thus, the purpose of the current study was to investigate some of the possible mechanisms underlying the protective effects produced by chronic sitagliptin treatment on pancreatic tissue in the ZDF rat, focusing on apoptosis, inflammation, angiogenesis and proliferation mediators.

## Methods

### Animals and experimental design

Male ZDF rats (ZDF/Gmi, *fa/fa*) and their littermates (ZDF/Gmi, *+/+*) were purchased from Charles River Laboratories (Barcelona, Spain) with 6 weeks of age and kept in our animal facility until they reached 20 weeks of age. Rats were properly housed, handled daily, and kept at a controlled standard temperature (22-23°C), humidity (60%) and light–dark cycles (12/12 h). Throughout the experiment, the animals were provided with distilled water *ad libitum* and rodent maintenance chow (A-04 Panlab, Barcelona, Spain, containing 15.4% of protein and 2.9% of lipids). The chow was adapted to the animal’s body weight (BW): 100 mg/g. Animal experiments were conducted according the European Council Directives on Animal Care and the National Laws. Along the text and in order to simplify the description of the animals, the ZDF/Gmi, *fa/fa* rats will be designated as diabetic rats, and, when under sitagliptin treatment, as sitagliptin-treated diabetic rats. The ZDF/Gmi, +/+ rats will be designated as lean control or control rats. The initial groups were established as 15 diabetic rats and as 10 lean control rats. When aged 20 weeks, n = 5 obese diabetic ZDF rats and the lean control (n = 5) were sacrificed for blood and tissue collection in order to establish the basal levels. The remainder lean control rats followed to week 26, as well as, the diabetic ZDF rats which were divided in two sub-groups (n = 5 rats each). The sitagliptin-treated group received by oral gavage, once a day (6:00 PM), during 6 weeks, 10 mg/kg/BW of sitagliptin dissolved in orange juice (vehicle) and the diabetic untreated group received, in the same conditions, only the vehicle (orange juice). The same procedures were adopted with the lean control rats. At 26 weeks of age, the animals were sacrificed by anaesthetic overdose, blood and tissues were collected for different analyses.

### Glycaemic, insulinaemic and lipidic profile assays

Serum glucose levels were measured using a Glucose oxidase commercial kit (Sigma, St. Louis, Mo, USA). Considering the variability of serum glucose levels in the rat, glycosylated haemoglobin (HbA1c) levels were used as an index of glucose control, through the DCA 2000+ latex immunoagglutination method (Bayer Diagnostics, Barcelona, Spain). Plasma insulin levels were quantified by using a rat insulin Elisa assay kit from Mercodia (Uppsala, Sweden). The steady state beta cell function of individual animals was evaluated using the previously validated homeostasis model assessment (HOMA) of β-cell function [31]. The formula used was as follows: [HOMA-β%] = 360 × fasting serum insulin (mU/L)/ fasting serum glucose (mg/dl) − 63. The values used (insulin and glucose) were obtained after an overnight of food deprivation. Serum triglycerides (TGs) were analysed on a Hitachi 717 analyser (Roche Diagnostics) using standard laboratorial methods. TGs kit was obtained from bioMérieux® (Lyon, France).

### Endocrine and exocrine pancreas lesions analysis by histopathology

#### Sample collection and preparation

The pancreas was immediately removed, placed in ice-cold Krebs’ buffer and carefully cleaned of extraneous fat and connective tissue; then the organ was cross-sectioned, fixed and processed for paraffin embedding in compliance with the histological protocols used.

#### Hematoxilyn & eosine staining

Samples were fixed in Bock’s fixative, embedded in paraffin wax and 3 μm thick sections were stained for routine histopathological diagnosis with haematoxylin and eosin (HE). All samples were examined by light microscopy using a Microscope Zeiss Mod Axioplan 2. Image acquisition was performed with digital microscope camera (Leica DFC450) and image processing was performed with the LAS Advanced Analysis Software Bundle (No: 12730448.). The degree of injury visible by light microscopy was scored in a double blind fashion. *Assessment of mean islet number*: Islets were counted using a 10× magnification, in three different microscopic fields and, the mean number per field was calculated for each study group. *Assessment of islet size*: Islet dimensions were obtained by measuring its maximum girth with an ocular grid of 1000 μm, using a 10× magnification. The maximum diameter was found by comparing all available radii diameters of each islet, and choosing the greatest. Islets were evaluated in three different microscopic fields and the mean size of the islets from each group of rats was calculated.

#### Histopathology

Appreciation of islet architecture was based on the uniformity of islet boundaries and classified as regular or irregular. Endocrine pancreatic damage was assessed by evaluating changes in the islets of Langerhans, namely, islet architecture (shape), presence of inflammatory infiltrate, fibrosis, and vacuolization and intra-islet congestion. A semi-quantitative rating for each slide ranging from 0 (minimal) to 3 (severe and extensive damage) was assigned to each of the studied components: inflammatory infiltrate, congestion, vacuolization and fibrosis. Each islet was examined and scored. Severity was graded as 0 = absent, 1 = mild, 2 = moderate and 3 = severe. Extension was evaluated by the area occupied by the lesion, an area of < 25% of the islet, was scored as 0, an area 25 - 50% scored as 1, an area 50 - 75% scored as 2, and if detected in an area > 75% scored as 3. The final score of each sample was obtained by the average of scores observed in individual islets. Exocrine pancreatic damage was evaluated according to the presence of congestion, fibrosis and inflammatory infiltrate in the interstitial tissues and graded also, by the same semiquantitative method, considering the entire exocrine parenchyma on the slide, as previously described [29].

#### Periodic acid of schiff staining

Periodic Acid of Schiff (PAS) was used to confirm the levels of islet and exocrine fibrosis. Samples were fixed in neutral formalin 10%, embedded in paraffin wax and 3 μm thick sections were immersed in water and subsequently treated with a 1% aqueous solution of periodic acid, then washed to remove any traces of the periodic acid and finally treated with Schiff’s reagent. A semi-quantitative rating was set for intensity and extension of staining, ranging from 0 (no staining) to 3 (intense and extensive staining).

### Pancreatic protein expression by immunohistochemistry

Formalin-fixed and paraffin embedded tissues were cut into 3 μm sections and deparaffinised in xylene. 3% H_2_O_2_ was used to remove endogenous peroxidase, and citrate buffered saline (pH 6.0), in MO, was used for antigen retrieval. Sections were preincubated with normal rabbit serum to prevent nonspecific binding and then incubated overnight at 4°C with anti-Bax (∆ 21, 1:50, rabbit polyclonal antibody, Santa Cruz, Biotechnology), Bcl2 (C 21, 1:50, rabbit polyclonal antibody, Santa Cruz, Biotechnology) and TRIB3 (ab22107, 1:100, rabbit polyclonal antibody, Abcam). The sections were then sequentially incubated at room temperature, with labelled-(strept)avidin-biotin-peroxidase method (LAB-SA) (Histostain®-Plus kit Zymed). Negative controls were included in each staining series, by omission of the primary antibodies. Positive controls were, respectively for Bax, Bcl2 and TRIB3 canine tonsils, canine breast carcinoma and the rat exocrine pancreas. Sections were counterstained with hematoxylin. The results were examined by light microscopy using a Zeiss Axioplan 2 microscope. Image acquisition and processing was performed according to described in the previous section. Immunopositivity was scored in accordance to staining intensity (I) and percentage of positive cells (P). Staining intensity was evaluated as 0, undetectable; 1, weak staining; 2, moderate staining and 3, intensive staining. Positive cells were evaluated in all Islets’ of Langerhans present on the slide. Final scoring for each rat was calculated by the Quick Score (Q) in which the percentage of positive cells (P) is multiplied by the intensity (I), using the formula: Q = P × I, resulting in a score between 0 – 300 [32]. The final score for each group was found by mean average.

### Pancreatic gene (mRNA) expression analysis by RT-qPCR

#### Sample collection and preparation

The pancreas were immediately collected, placed in ice-cold Krebs buffer for cleaning of collective tissue and immediately frozen at −80°C in preservative RNA later™ solution (Ambion, Austin, TX, USA) until analysis. Gene (mRNA) expression was evaluated by real-time RT-qPCR for markers of apoptotic machinery, inflammation and proliferation/angiogenesis.

#### Total RNA isolation

Samples were removed from the RNA later™ preservation solution and 1200 μL of RLT Lysis Buffer was added to proceed with disruption and homogenization for 2 min at 30 Hz using TissueLyser (Qiagen, Hilden, Germany). Tissue lysate were processed according to the protocol from RNeasy® Mini Kit (Qiagen, Hilden, Germany). Total RNA was eluted in 50 μL of RNase-free water (without optional treatment with DNAse). In order to quantify the amount of total RNA extracted and verify RNA integrity (RIN, RNA Integrity Number), samples were analysed using 6000 Nano Chip® kit, in Agilent 2100 bioanalyser (Agilent Technologies, Walbronn, Germany) and 2100 expert software, following manufacturer instructions. The yield from isolation was from 0.5 to 3 μg; RIN values were 6.0-9.0 and purity (A_260_/A_280_) was 1.8-2.0.

#### Reverse transcription

RNA was reverse transcribed with SuperScript™ III first-strand synthesis system for RT-PCR (Invitrogen, California, USA). One microgram of total RNA was mixed with a 2× First-Strand Reaction Mix and a SuperScript™ III Enzyme Mix (Oligo(dT) plus Random hexamers). Reactions were carried out in a thermocycler Gene Amp PCR System 9600 (Perkin Elmer, Norwalk, CT, USA), 10 min at 25°C, 50 min at 50°C and 5 min at 85°C. Reaction products were then digested with 1 μL RNase H for 20 min at 37°C and, finally, cDNA eluted to a final volume of 100 μL and stored at −20°C.

#### Relative quantification of gene expression

Performed using 7900 HT Sequence Detection System (Applied Biosystems, Foster City, CA, USA). A normalization step preceded the gene expression quantification, using geNorm Housekeeping Gene Selection kit for Rattus norvegicus (Primer Design, Southampton, UK) and geNorm software (Ghent University Hospital, Center for Medical Genetics, Ghent, Belgium) to select optimal housekeeping genes to this study [33]. Real-time PCR reactions used specific QuantiTect Primer Assays (Qiagen, Hilden, Germany) with optimized primers for Bax, Bcl2, TRB3, IL-1β, PCNA and VEGF. Endogenous controls were also used: GAPDH, ACTB, TOP1, and RPL13 together with QuantiTect SYBR Green PCR Kit Gene expression (Qiagen, Hilden, Germany) according to manufacturer’s instructions. RT-qPCR reactions were carried out with 100 ng cDNA sample, primers (50–200 nM) and 1X QuantiTect SYBR Green PCR Master Mix. Nontemplate control reactions were performed for each gene, in order to assure no unspecific amplification. Reactions were performed with the following thermal profile: 10 min at 95°C plus 40 cycles of 15 s at 95°C and 1 min. at 60°C. Real-time PCR results were analyzed with SDS 2.1 software (Applied Biosystems, Foster City, CA, USA) and quantification used the 2^−ΔΔCt^ method [34].

### Statistical analysis

For all biochemical measurements made over time and treatment effect, independent samples t-Student test was used. For histopathology and immunohistochemistry data: Chi-square test with Monte Carlo simulation or exact test (when contingency tables are 2×2) was performed to find out the differences in lesions of endocrine/exocrine pancreas between lean control and diabetic ZDF rats at the beginning of the study, (20 week-old); untreated and sitagliptin treated diabetic ZDF and lean control rats at 26 weeks of age. Independent samples t-Student test was used to determine the differences in the number, regularity and size of the pancreatic islets between lean control and diabetic ZDF rats in the pre-therapeutic stage, at 20 weeks; untreated and sitagliptin-treated diabetic ZDF and lean control ZDF rats at 26 weeks of age. Data were analysed using SPSS Statistics 20 (2011). For RT-qPCR data: For statistical analysis, we used the GraphPad Prism, Version 5.0. Comparisons between groups were performed using ANOVA and the post-hoc Bonferroni test. All values are reported as mean ± SEM (standard error of means). Significance level was accepted at 0.05.

## Results

### Sitagliptin prevents aggravation of glycaemic, insulinaemic and lipidic profiles

Concerning body weight, no significant differences were encountered between the diabetic and the lean control rats at the beginning of treatments (T0: week 20), despite the obese profile encountered in the diabetic rats between the 8th and the 14th week (data not shown). At the end of the study (26 weeks), the untreated diabetic rats exhibited a reduction in their BW (p < 0.001); nevertheless, the lean control group gained weight. Sitagliptin treatment, during 6 weeks, stabilized the loss of weight in the diabetic ZDF rats, even preventing part of the BW loss when compared with the rats without treatment (Table 1).

**Table 1:** Body weight and glycaemic, insulinaemic and lipidic profile in the rats under study at the initial and final times

**Time**	**Initial time (20 wks old rats)**		**Final time (26 wks old rats)**		
**Groups**	**Control**	**Diabetic**	**Control**	**Diabetic**	
**Parameters**	**Vehicle (n = 10)**	**Vehicle (n = 15)**	**Vehicle (n = 5)**	**Vehicle (n = 5)**	**Sitagliptin (n = 5)**
BW (g)	406.70 ± 6.83	388.10 ± 8.87	445.70 ± 8.16	354.40 ± 8.85^aaa^	380.00 ± 14.46
Glucose (mg/dl)	131.80 ± 1.20	512.00 ± 4.53^aaa^	133.40 ± 2.16	623.60 ± 7.30^bb^	520.44 ± 3.31^ccc^
HbA1c (%)	3.66 ± 0.15	9.96 ± 0.86^aaa^	3.76 ± 0.26	10.68 ± 0.75	8.68 ± 0.37^ccc^
Insulin (mU/ml)	14.82 ± 3.90	11.62 ± 1.15^aaa^	15.13 ± 3.34	7.16 ± 1.14^bbb^	11.19 ± 1.73^ccc^
HOMA-Beta (%)	77.70 ± 2.28	9.33 ± 0.83^aaa^	77.52 ± 1.10	4.60 ± 0.16^bbb^	8.82 ± 0.32^ccc^
TGs (mg/dl)	111.60 ± 8.33	366.00 ± 5.92^aaa^	158.40 ± 6.80	409.80 ± 8.04^bb^	255.60 ± 3.70^ccc^

Values are means ± SEM of n rats. Comparisons between groups: ^aaa^ = p < 0.001 between diabetic versus control groups (20 or 26 weeks); ^bb^ and ^bbb^ = p < 0.01 and p < 0.001, respectively, within the diabetic group (26 versus 20 weeks); ^ccc^ = p < 0.001 between diabetic sitagliptin-treated versus diabetic untreated groups (at 26 weeks).

At the beginning of treatments (T0: week 20), fasting blood glucose, HbA1c and TG were already significantly higher in diabetic rats when compared with their lean counterparts, indicative of a metabolic deregulation. These results were accompanied by a decrease in fasting serum insulin and in the functional ability of the pancreas demonstrated by the reduction of 87.99% in HOMA-beta values. An age-dependent increase in the metabolic deregulation was observed in diabetic untreated ZDF rats presenting augmented levels of glucose, HbA1c and TG from 20 to 26 weeks old. They also exhibit an aggravation of the relative insulinopaenia, as well as a decrease in estimated steady-state beta cell function (*p* < 0.001). Six weeks of sitagliptin treatment was able to significantly ameliorate all the metabolic parameters as shown in Table 1. In fact, sitagliptin significantly (*p* < 0.001) prevented the additional increase in blood glucose and serum TG contents (16.54% and 37.63%, respectively, vs untreated), while preventing further decrease in serum insulin and enhancing the functional capacity of beta cells (156.28% and 191.74%, respectively, vs untreated) (Table 1).

### Sitagliptin prevents aggravation of endocrine and exocrine pancreatic damage

**Figure 1:**
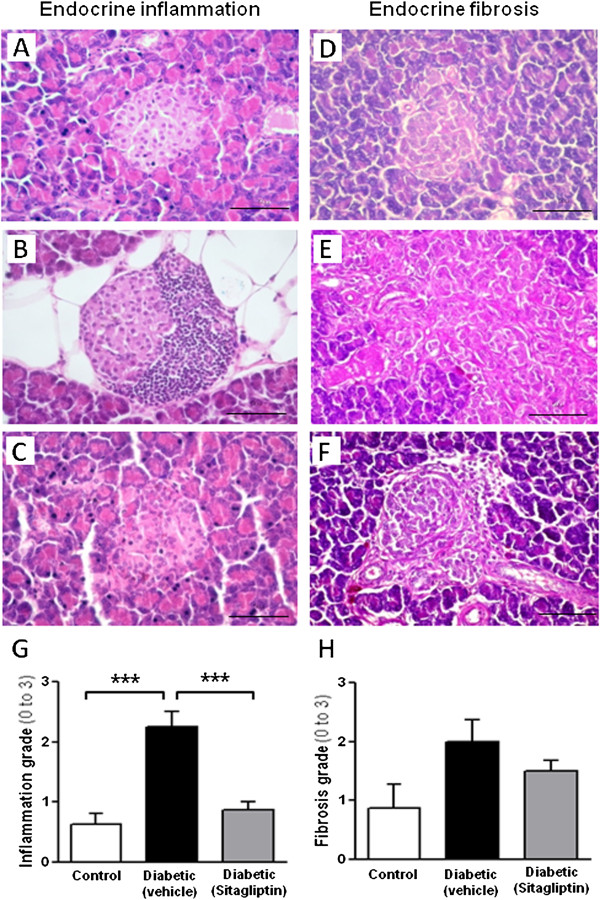
**Sitagliptin effects on inflammation and fibrosis of endocrine pancreas of diabetic ZDF rats.** Endocrine inflammation in 26 week-old animals (images **A**-**C**): **(A)** Micrograph of normal islet of Langerhans observed in a lean control ZDF rat; **(B)** Islet of Langerhans of an untreated diabetic ZDF rat showing severe inflammatory infiltrate occupying over 50% of the islet’s area (Grade 3 inflammation); **(C)** A clearly regression of inflammation is observed after 6 weeks of sitagliptin treatment in obese diabetic ZDF rat (Grade 1 inflammation). Endocrine fibrosis observed in 26 week-old animals (images **D**-**F**): **(D)** Normal islet of Langerhans of lean control ZDF rat; **(E)** Grade 3 fibrosis in an untreated obese diabetic ZDF rat, displaying a large and irregularly shaped islet filled with fibrous tissue, evidenced by intense pink staining; **(F)** Grade 1 fibrosis in a regular, small islet in sitagliptin treated diabetic ZDF rats. Semiquantitative evaluation of inflammation (graph **G**) and fibrosis (graph **H**): **(G)** A significantly higher (*p* < 0.001) endocrine inflammation in diabetic rats was recorded when compared to lean control animals. Sitagliptin treatment of diabetic ZDF rats during 6 weeks significantly reduced (*p* < 0.001) inflammation in comparison to untreated counterparts; **(H)** Data for endocrine fibrosis showed a trend for an increase in diabetic rats when compared to lean control animals, and a trend for improvement with sitagliptin treatment, although without statistical significance. Chi-square test with Monte Carlo simulation or exact test (when contingency tables are 2 × 2) was performed to find out the differences in histomorphological lesions observed in endocrine pancreas. (*p* < 0.05, *p* < 0.01 and *p* < 0.001 for one, two or three symbols, respectively; n = 5 per group).

Comparative analysis between the endocrine pancreas of lean control and diabetic ZDF rats of 20 weeks of age revealed a significant increase in inflammation and fibrosis of Langerhans islets in the diabetic group (*p* < 0.01), with no statistically significant differences in the other analyzed parameters (data not shown). At 26 weeks of age, endocrine pancreatic inflammation was significantly higher (*p* < 0.001) in the diabetic rats when compared with the lean control animals (Figure 1A, B and G). Sitagliptin-treated diabetic ZDF rats showed significantly (*p* < 0.001) reduced inflammation when compared with the untreated diabetic rats (Figure 1B, C and G). A similar profile was encountered concerning endocrine pancreatic fibrosis, which, despite the differences, did not reach statistical significance (Figure 1D-H).

**Figure 2:**
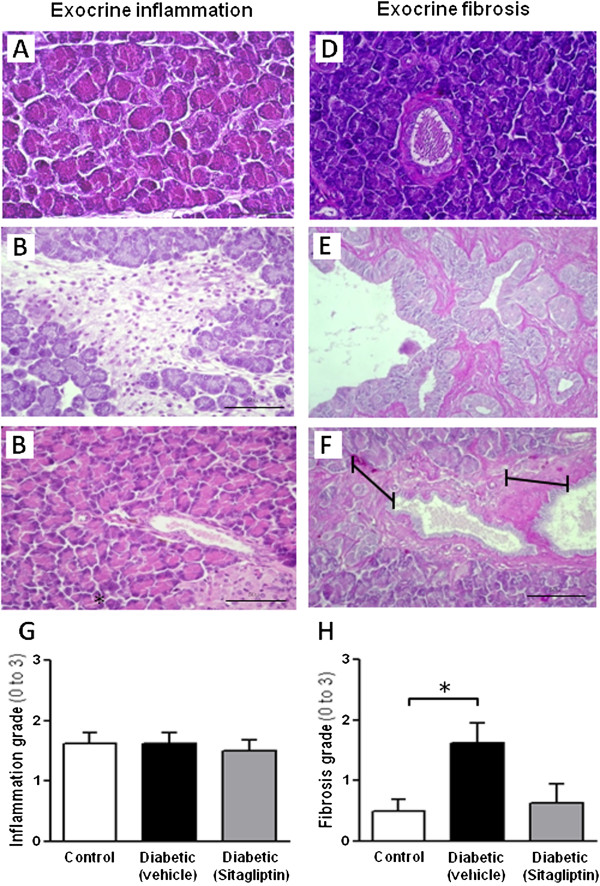
**Sitagliptin effects on inflammation and fibrosis of exocrine pancreas of diabetic ZDF rats.** Histopathological observation of exocrine inflammation in 26 week-old animals (images **A**-**C**): **(A)** Micrograph of a normal exocrine parenchyma of lean control ZDF rat; **(B)** The untreated diabetic rats exhibiting severe inflammatory infiltrate, amid the acini of the exocrine parenchyma (grade 3 inflammatory lesions); **(C)** Normal exocrine parenchyma with no inflammatory signs in the exocrine pancreas of sitagliptin-treated diabetic ZDF rats. Histopathological observation of exocrine fibrosis in 26 week-old animals (images **D**-**F**): **(D)** Normal pancreatic duct in a lean control ZDF rat; **(E)** An extremely thickened, grade 3, fibrotic duct, which overextends the microscopic field (interrupted line), with numerous *neocanaliculi* in the duct wall, .present in an untreated diabetic ZDF rat; **(F)** Improvement of duct fibrosis from grade 3 to grade 1 (full line) in a 6 weeks sitagliptin treated diabetic ZDF rat. Semiquantitative evaluation of inflammation (graph **G**) and fibrosis (graph **H**): **(G)** Exocrine pancreatic inflammation revealed only a slight descent in sitagliptin treated animals in relation to its untreated counterparts; **(H)** Sitagliptin presented a trend to decrease the duct fibrosis increment (p < 0.05) found in the untreated ZDF rats. Chi-square test with Monte Carlo simulation or exact test (when contingency tables are 2×2) was performed to find out the differences in histomorphological lesions observed in exocrine pancreas (*p* < 0.05, *p* < 0.01 and *p* < 0.001 for one, two or three symbols, respectively; n = 5 per group).

Concerning the exocrine pancreas lesions, in rats aged 26 week-old, while no significant changes were found concerning exocrine pancreas inflammation (Figure 2A-C and G), fibrosis was significantly (*p* < 0.05) increased in the diabetic ZDF rats, when compared with the lean control, which was prevented in the diabetic animals under sitagliptin therapy (Figure 2D-H).

### Cytoprotective effects of sitagliptin against pancreatic damage progression

**Figure 3:**
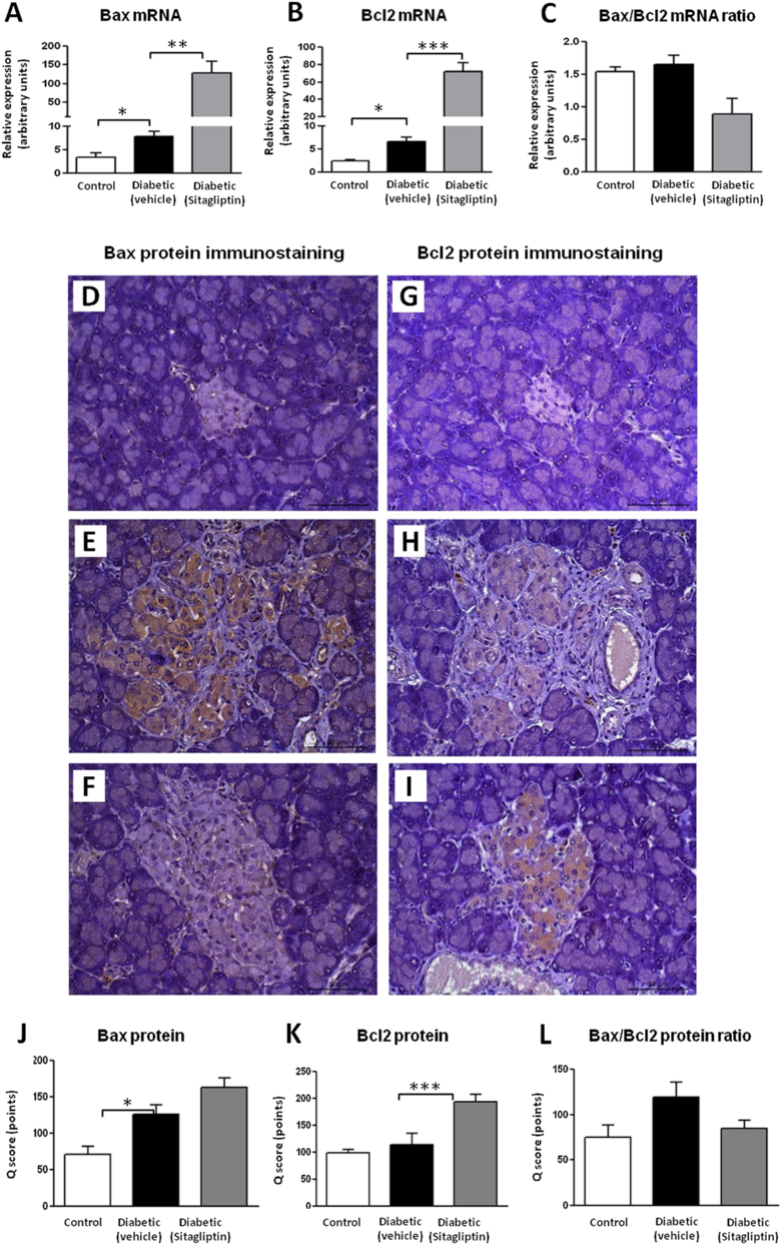
**Sitagliptin protects the diabetic ZDF rats against endocrine pancreas apoptotic cell death.** Upper panel **(A-C)** - pancreas mRNA expression of Bax and bcl2 in 26 week-old ZDF rats: A significant increase (p < 0.05) in apoptotic protein Bax **(A)**, as well as, in antiapoptotic Bcl2 **(B)** was observed in the untreated diabetic ZDF rats when compared with the lean control ZDF animals, resulting in an unchanged Bax/Bcl2 ratio **(C)**. In the sitagliptin-treated diabetic rats an overexpression of the mRNA was registered for both Bax (not statically significant) and Bcl2 (*p* < 0.001) ensuing a reduced Bax/Bcl2 ratio **(C)**. Middle panel **(D-I)** - Immunostaining of pancreas Bax and Bcl2 in 26 week-old ZDF rats: **(D)** The expression of Bax protein is not present in the pancreatic islet (grade 0) of a lean control rat; **(E)** A deeply stained islet (grade 3) revealing Bax protein expression in untreated diabetic ZDF rat; **(F)** The diabetic sitagliptin-treated rats displayed a light stained islet (grade 1); **(G)** Expression of Bcl2 protein not observed in islet (grade 0) of a lean control rat; **(H)** A moderately stained islet (grade 2) with Bcl2 antibody in an untreated diabetic ZDF rat; **(I)** An intensely stained islet (grade 3) in a diabetic sitagliptin-treated rat. Lower panel **(J-L)** - Quantification of protein expression: Bax protein expression in diabetic untreated rats exhibited a significant increase in relation to lean control rats; in sitagliptin-treated diabetic rats, Bax presented a trend for overexpression **(J)**, accompanied by a significantly (*p* < 0.001) increased expression of Bcl2 **(K)**, resulting in a Bax/Bcl2 ratio identical to control animals **(L)**. Statistical comparisons between groups were performed using one-way ANOVA and the *post-hoc* Bonferroni test (*p* < 0.05, *p* < 0.01 and *p* < 0.001 for one, two or three symbols, respectively; n = 5 per group).

Pancreatic tissue mRNA levels of mediators of apoptotic machinery showed a significantly increased (*p* < 0.05) expression of the apoptotic Bax, as well as, antiapoptotic Bcl2 in the 26 week-old diabetic ZDF rats (Figure 3A and B, respectively), when compared with the lean ZDF animals, thus resulting in an unchanged Bax/Bcl2 ratio (Figure 3C). In the diabetic rats under sitagliptin treatment, there was an overexpression of the mRNA for both Bax and Bcl2, favouring a reduced Bax/Bcl2 ratio (Figure 3A-C) as a result of a higher increment of mRNA expression of Bcl2 when compared with Bax.

The pancreatic mRNA expression of Bax and Bcl2 was accompanied by protein expression studies of immunohistochemistry (Figure 3D-L). In the untreated diabetic animals there was a significantly (*p* < 0.05) rise in Bax stained cells and unchanged Bcl2, resulting in a trend to an increased Bax/Bcl2 ratio, when compared with the controls; sitagliptin-treated diabetic rats presented a trend for increased protein expression of Bax, accompanied by a significantly (*p* < 0.001) increased expression of Bcl2, which results in a Bax/Bcl2 ratio identical to that found for the control animals (Figure 3D-L).

**Figure 4:**
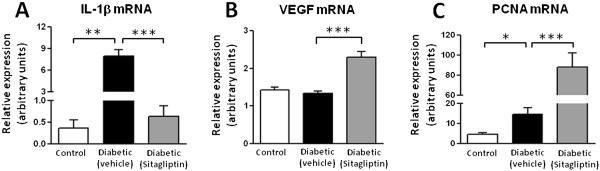
**Effects of sitagliptin treatment on pancreatic mRNA expression of mediators of inflammation, angiogenesis and proliferation: IL-1β, VEGF and PCNA. (A)** mRNA expression of pancreatic IL-1β in untreated diabetic ZDF rats was significantly increased (*p* < 0.01) in contrast to lean control rats; in the sitagliptin-treated diabetic group, overexpression of IL-1β was significantly prevented (*p* < 0.001). Overexpression (*p* < 0.001) of VEGF **(B)** and PCNA **(C)** mRNA in ZDF diabetic rats treated with sitagliptin for 6 weeks when compared with untreated diabetic rats. Statistical comparisons between groups were performed using one-way ANOVA and the *post-hoc* Bonferroni test (*p* < 0.05, *p* < 0.01 and *p* < 0.001 for one, two or three symbols, respectively; n = 5 per group).

**Figure 5:**
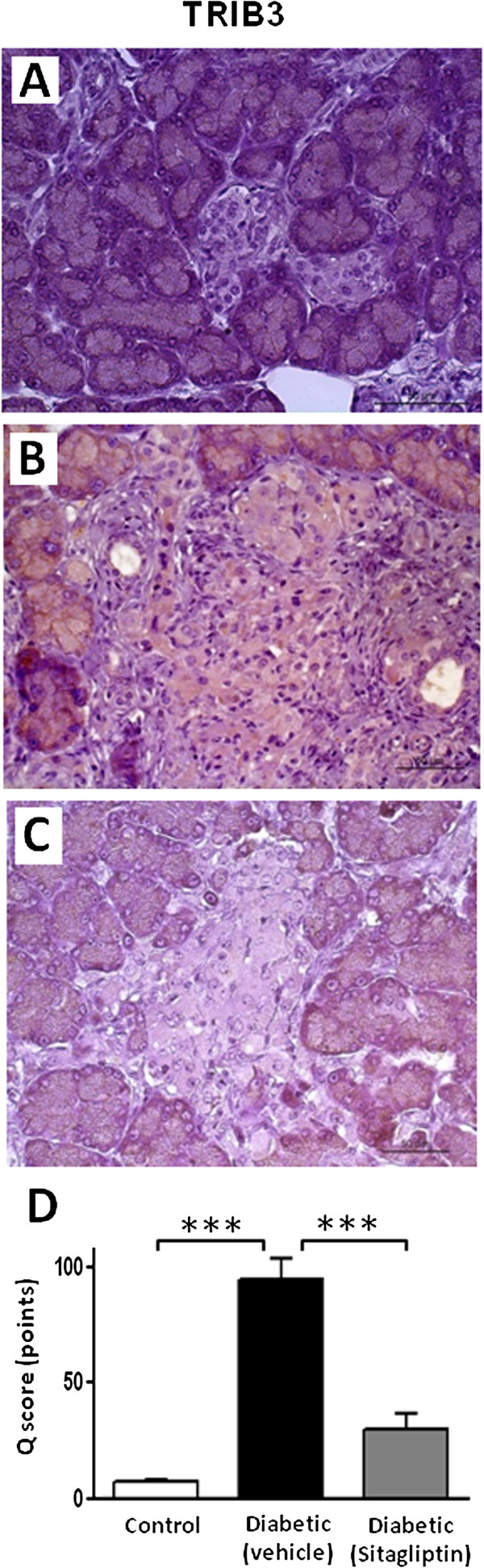
**Sitagliptin prevents TRIB3 protein overexpression in pancreas of ZDF diabetic rats.** Micrographs of TRIB3 expression by immunostaining in 26 week-old ZDF rats **(A-C)**: **(A)** Lean ZDF control rat presenting unstained pancreatic islet (grade 0); **(B)** Untreated diabetic ZDF rat showing an intensely stained pancreatic islet (grade 3); **(C)** Sitagliptin-treated diabetic rat for 6 weeks displayed light staining of endocrine cells (grade 1). Exocrine pancreatic tissue presents normal constant staining for TRIB3 in all three groups. **(D)** Quantification of protein expression by score points: expression of TRIB3 is almost undetectable in control rats, rising very significantly (*p* < 0.001) in untreated diabetic ZDF rats; and declining very meaningfully (*p* < 0.001) in 6 weeks sitagliptin-treated diabetic treated rats. Statistical comparisons between groups were performed using one-way ANOVA and the *post-hoc* Bonferroni test (*p* < 0.05, *p* < 0.01 and *p* < 0.001 for one, two or three symbols, respectively; n = 5 per group).

Concerning other putative mechanisms behind the protective effects of sitagliptin on the pancreatic tissue, we found that the diabetic rats, aged 26 weeks, presented a significantly increased pancreatic mRNA expression of IL-1β (*p* < 0.01), which was prevented (*p* < 0.001) in the sitagliptin-treated group (Figure 4A). Sitagiptin was able to promote overexpression (*p* < 0.001) of VEGF and PCNA mRNA when compared with the untreated diabetic rats (Figure 4B and C, respectively). In addition, sitagliptin treatment totally (*p* < 0.001) prevented the diabetes-induced increment (*p* < 0.001) in TRIB3 expression in the pancreatic tissue (Figure 5A-D).

## Discussion

Previous studies propose that a disruption of the normal relationship between insulin sensitivity and pancreatic β-cell function is crucial for the pathogenesis of T2DM [4], and that the degeneration of Langerhans islets with β-cell loss is secondary to insulin resistance and may have a key role in the progression of the disease [5,6]. Furthermore, the loss of β-cell mass is not yet completely elucidated, but a possible cause may reside in apoptotic processes and in a lost capacity for pancreatic regeneration [28,35,36]. Previous studies have been suggesting that gliptins are able to preserve both β-cell function and cell mass in animal models of diabetes [29,37-39], but the mechanisms underlying the protective effects remain to be elucidated.

Consistent with previous reports our study demonstrated that a 6 weeks sitagliptin treatment was able to improve β-cell function as well as preserve pancreatic islet structure. We hypothesize that sitagliptin is able to preserve pancreatic function by improving insulin resistance and by other cytoprotective properties, including antiapoptotic, anti-inflammatory and pro-proliferative, based on the cytoprotective properties previously reported for incretin peptides in distinct tissues [29,37-43]. In fact, the results presented herein strongly suggest that in diabetic ZDF rats sitagliptin may derive its cytoprotective effects via two different type of influences: directly reducing apoptosis and promoting cell proliferation due to increase incretin availability; indirectly via metabolic effects, including amelioration of chronically elevated glucose and triglycerides, prevention of insulinopaenia and reduction of inflammation, thus protecting from deleterious effects derived from glucotoxicity, lipotoxicity and insulin resistance.

The histomorphological evaluation of endocrine and exocrine pancreatic tissue shows that the differences between diabetic untreated and sitagliptin-treated animals were striking. In fact, the sitagliptin-treated rats presented an amelioration of inflammation and fibrosis in endocrine and exocrine pancreas. In particular, inflammation was highly reduced in the islets of Langerhans, and the exocrine pancreas of diabetic rats receiving sitagliptin did not present fibrotic changes in the vascular and the ductal walls. The changes described above were repeatedly and systematically observed by two pathologists unaware of the identity of the slides. These findings are in accordance with our preliminary work [29] but in contradiction with the results obtained by Matveyenko et al. [37] using a DPP IV inhibitor in human IAPP transgenic (HIP) rats and by Nachnani et al. [41] using an injection of GLP-1 agonist, who suggest that the enhancement of endogenous GLP-1 levels could induce undetected low grade asymptomatic chronic pancreatitis. The histomorphological observations were in accordance with an improvement in pancreatic beta-cell function as shown by the augmentation in HOMA-beta in diabetic sitagliptin-treated rats. The effects of chronic inhibition of DPP-IV in increasing β-cell mass and function over time could be due, at least in part, by the increase in glucose-stimulated insulin secretion, which is believed to be mediated primarily via stabilization of the incretin hormones, including GLP-1 [27].

It is well established that apoptosis is one of the pathways responsible for the progressive deterioration of beta cell and evolution of diabetes. Our study suggests that sitagliptin is able to promote an antiapoptotic effect, which is in agreement with other reports [37-40,44] in the pancreatic tissue. In fact, Matveyenko et al. [36] reported that sitagliptin therapy led to preservation of β-cell mass in HIP rats as compared with its untreated counterparts, while Maida et al. [38] reported an increment of percentage of β-cell area in streptozotocin-induced diabetic mice under sitagliptin treatment. The antiapoptotic properties of sitagliptin is also in agreement with the effects reported in extra-pancreatic tissues, such as the kidney, with improvement of renal function and reduction of parenchymal damage, due to a decrease in apoptosis, inflammation and an increase of in antioxidant capacity [30,40,45,46]. Apart from the antiapoptotic effect suggested by our results, the protective effects afforded by sitagliptin on the pancreas tissue might be the result of other activities previously described for the incretin peptides, including GLP-1 [15,16,47-51]. Anti-inflammatory, pro-angiogenic and pro-proliferative properties are suggested by the reduced expression of IL-1β and by the increased expression of VEGF and PCNA observed in the pancreas tissue of sitagliptin-treated diabetic rats. Inflammation has been associated with the development of insulin resistance, beta cell apoptosis and evolution of diabetes, and IL-1β is one of the main inflammatory cytokines in the process [52,53]. We and other authors have suggested anti-inflammatory properties of gliptins in distinct animal models and tissues, as well as, in type 2 diabetic patients [29,43,45,54-56], in agreement with our hypothesis. VEGF is expressed in the endocrine cells and the increased VEGF expression found in the diabetic rats under sitagliptin treatment might be viewed as an increased capacity for tissue regeneration. The same is true for PCNA, which is an indicator for cell proliferation and has been used in the present work to determine β-cell mass expansion [57,58]. It could be hypothesized that sitagliptin-evoked increased GLP-1 availability, due to inhibition of its degradation by DPP-IV, will favour the development of new cells, via proliferation enhancement of pre-existing cells and induction of islet neogenesis, effects that were previously reported for GLP-1 [27].

The second mechanism involved in the effect of sitagliptin may be related to significant improvement in the metabolic profile, including amelioration of glucose, insulin and TGs levels. We must empathize that the dose of sitagliptin used in our study (10 mg/kg/day) may be considered a low dose as others have used higher doses or a twice a day treatment [37,38,40]. Nevertheless, sitagliptin treatment improved hyperglycaemia and hypertriglyceridaemia, thus ameliorating glucolipotoxicity in the diabetic ZDF rats. Several pathophysiological mechanisms have been identified as potential contributors to β-cell stress and subsequent dysfunction, including glucotoxicity, lipotoxicity, and increased secretory demand resulting from insulin resistance. In addition, disturbances in secretion of various adipose tissue-secreted factors or cytokines derived from the innate immune system might also play a causal role [18]. Furthermore, both hyperglycaemia and hyperlipidaemia are associated with induction of oxidative stress in β-cells. Previously, we have already demonstrated using this animal model that a low-dose chronic sitagliptin treatment was able to promote a favourable impact on chronic inflammation and oxidative stress [29]. In this context, it should also be noted that the effect of liraglutide upon endoplasmatic reticulum stress, oxidative stress and cell apoptosis in diabetic *db/db* rats, as well as the results of vildagliptin in diabetic KK-A^*y*^ mice, are essentially compatible with those observed in this study [59,60].

Malfunctioning insulin secretion and/or insulin resistance are recognized as key factors for the pathogenesis of T2DM [61,62]; the latter results from anomalies in the insulin signaling cascade, a regulated complex molecular pathway, which may be inhibited and activated by many biochemical mechanisms [62]. One of the genes implicated in coding inhibitors of insulin signaling and action is TRIB3, a mammalian tribbles homolog that binds Akt inhibiting downstream insulin-signalling cascade [62,63]. Our current study revealed that 26 week old ZDF diabetic rats showed pancreas overexpression of TRIB3 which, concurrently, showed insulin resistance and relative insulinopaenia. Sitagliptin treatment was able to completely reduce tissue TRIB3 expression, which might be a key mechanism for the decline of insulin resistance and improvement of insulin secretion observed in the diabetic rats under sitagliptin treatment. It has been shown, in cellular and animal models, that changes in TRIB3 expression levels induce systemic insulin resistance [64-66]. Indeed, increased TRIB3 expression was observed in islets from T2DM donors and high fed diet (HFD) mice [61,63]. In humans, TRIB3 has also been associated with insulin resistance and T2DM, accompanied by enhanced inhibition of insulin signalling and AKT/PKB activation in different tissues, including the β cells [61,67,68]. Prior rodent studies [64-66,69], indicate that TRIB3 overexpression plays a major role in modulating whole-body insulin sensitivity and suggest a possible involvement in the pathogenesis of insulin resistance-related metabolic abnormalities. Another pivotal aspect by which TRIB3 seems to be associated with the evolution of insulin resistance and pancreas degradation is its role in inducing apoptosis in pancreatic β-cells and inhibiting cell proliferation; so, by downregulating the expression of TRIB3, sitagliptin promotes antiapoptotic effects and enhance β-cell proliferation, thus contributing to the beneficial effects afforded by this DPP-IV inhibitor in this animal model.

## Conclusions

In this animal model of obese type 2 diabetes (the ZDF rat) sitagliptin prevented β-cell dysfunction and evolution of pancreas damage. The protective effects afforded by this DPP-IV inhibitor may derive from improvement of metabolic profile (related to amelioration of glycaemic and lipidic levels and of insulin resistance) and from cytoprotective properties. In fact, sitagliptin was able to reduce Bax/Bcl2 ratio, suggestive of an antiapoptotic effect, and completely prevented the increased pancreas overexpression of IL-1β and TRIB3 found in the untreated diabetic animals, thus demonstrating an anti-inflammatory action; in addition, sitagliptin was able to promote overexpression of VEGF and PCNA, suggesting pro-angiogenic and pro-proliferative properties. From a therapeutic viewpoint, our results reinforce the status of sitagliptin as a promising antidiabetic drug not only by the expected glycaemic control but also, and specially, by the ability to prevent the decline of insulin secreting capacity in pancreatic islets through tissue-cytoprotective properties, thus suggesting a role in the prevention of diabetes evolution.

## Abbreviations

BW: Body weight; CRP: C-reactive protein; DPP-IV: Dipeptidyl peptidase IV; GIP: Gastric inhibitory polypeptide; GLP-1: Glucagon-like peptide-1; HbA1c: Glycosylated haemoglobin; HE: Haematoxylin and eosin; HFD: High fed diet; HIP: Human IAPP; HOMA: Homeostasis model assessment; IL-1β: Interleukin 1 beta; PAS: Periodic acid of Schiff; PCNA: Proliferating cell nuclear antigen; RT-qPCR: Reverse transcription quantitative polymerase chain reaction; SEM: Standard error of means; T2DM: Type 2 diabetes mellitus; TGs: Triglycerides; TNF-α: tumor necrosis factor alpha; VEGF: Vascular endothelial growth factor; WHO: World Health Organization; ZDF: Zucker Diabetic Fatty.

## Competing interests

The authors declare that they have no competing interest.

## Authors’ contributions

CM, FT, RF, FR and ETL conceived and designed the study protocol. CM, HV, PR-S, RF, FR and ETL participated in the animal handling and biochemical laboratory assays. HV and PR-S coordinated the histopathological and gene expression studies, respectively. CM, HV, JO, FR and ETL analyzed the data. CM, JO, RF, FR and ETL prepared the manuscript. FR and ETL equally contributed to the work and are co-last authors and co-responsible for the work and manuscript. All authors read and approved the final manuscript.
